# A focus on sustainable energy management for self-powered IoT devices *via* indoor photovoltaics

**DOI:** 10.1039/d3sc90109b

**Published:** 2023-06-12

**Authors:** Pablo Docampo

**Affiliations:** a School of Chemistry, University of Glasgow University Place G12 8QQ Glasgow UK pablo.docampo@glasgow.ac.uk

## Abstract

The future of information technologies lies in the form of trillions of autonomous ‘smart objects’ that can sense and communicate with their environment delivering pervasive and ubiquitous computing beyond today's imagination. Michaels *et al.* (H. Michaels, M. Rinderle, I. Benesperi, R. Freitag, A. Gagliardi and M. Freitag, *Chem. Sci.*, 2023, 14, 5350, https://doi.org/10.1039/D3SC00659J) have achieved a key milestone in this context by developing an integrated autonomous and light-powered Internet of Things (IoT) system. They also show that dye-sensitized solar cells are particularly well-suited for this purpose with an indoor power conversion efficiency of 38%, far surpassing conventional silicon photovoltaics and alternative indoor photovoltaics technologies.

## Abstract 2

Michaels *et al.* (*Chem. Sci.*, 2023 https://doi.org/10.1039/D3SC00659J) show how employing a dye combination that matches the indoor light spectrum maximises the efficiency of dye cells to power autonomous Internet of Things systems.

## Graphical abstract

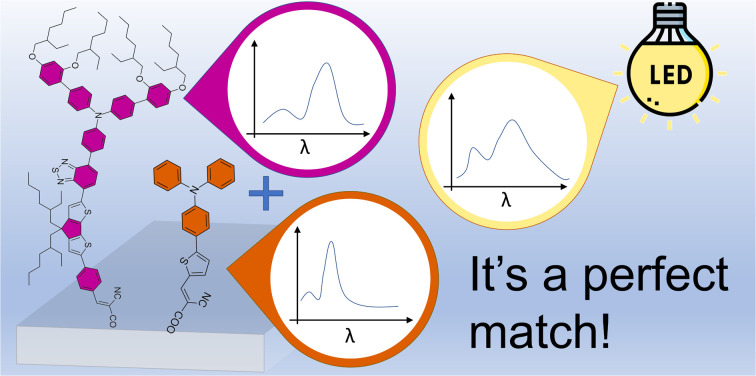

To effectively enable production at the trillions scale, next-generation devices must be compatible with low-cost, mass-production techniques such as printed electronics, and must employ sustainable materials. At the same time, devices must limit power consumption to stave off exponential energy consumption growth rates while requiring no maintenance to be effective in their deployment. To achieve this, the central design paradigm requires integrated and autonomous systems that scavenge energy sources available locally. In this context, no renewable energy source is more abundant or cheaper than light, prompting a large body of researchers to develop solutions to harvest it as efficiently as possible. Yet, traditional inorganic semiconductor-based photovoltaics suffer from low power conversion efficiencies under white LED indoor artificial light and are typically rigid,^1^ making their implementation a challenge.

Taking advantage of light as a power source brings in a second challenge from the computing side since a stable power source is at the core of design practices. What this looks like in practice is that autonomous Internet of Things (IoT) devices typically employ a battery which ultimately requires upkeep as it needs to be replaced. In order to delay this as much as possible, the focus has been on maximising the energy efficiency of existing hardware through low-power protocols, although these bring their own challenges in turn as a design targeting minimum power (energy) severely degrades performance and energy efficiency (peak power consumption)^2^ or requires the incorporation of additional energy management circuitry.^3^ Significant advances in the development of network protocols that minimise power usage, *i.e.* LoRa, BLE, *etc.*^4,5^ have significantly enhanced battery life. Yet, no matter the approach, at some point the battery will need to be replaced, representing a clear and considerable barrier to more widespread development. Incorporating a light harvester in the form of a photovoltaic solar cell has the potential to address this issue and capitalise on this emerging market.^2^ Yet, one cannot simply replace a battery in conventional circuits with a solar cell and expect a working solution; a suitable interface needs to be developed to cope with the variable nature of the power source.

Michaels *et al.* have tackled the latter challenge in a very interesting way (https://doi.org/10.1039/D3SC00659J).^6^ Firstly, they have implemented a machine learning algorithm based on long short-term memory (LSTM)^7^ to predict the available power for computation based on the previous 12 hours timeframe. This enables the device to adapt to the changing light environment, *e.g.* when sunlight through a window is available in a home environment. This is a significant departure from current approaches, clearly allowing a much wider spread of tasks based on the available power rather than working to minimise computational tasks to minimise power consumption, and is a more effective use of the available energy. Following on this theme, the team developed an interface between the solar cells and the device that employs two supercapacitors and a voltage divider. This activates the ‘wake-up’ part of the cycle only when a certain amount of energy is stored in the capacitors, effectively ‘hardwiring’ the adaptive response to available energy in the system and enabling the system to adapt to changing light conditions.

The second challenge addressed by Dr Freitag's team is that of maximising the available energy in indoor conditions. The light intensity available from artificial lighting is much lower than that of sunlight, so typical devices optimised for outdoor power generation are generally much less efficient in indoor environments. This is particularly true for traditional inorganic semiconductor-based photovoltaics which are typically more efficient when under higher light bias, *i.e.* sunlight or solar concentrator arrays, *etc.*^8^ but can lose up to two-thirds of the performance in indoor conditions.^9^ The reverse is true for dye-sensitized solar cells which typically suffer from ion-diffusion limitations at higher light intensities, *i.e.* ions must physically move from one end of the device to the other, with effects more pronounced for modern, bulkier redox shuttles.^10,11^

Yet, these limitations disappear under typical indoor lighting conditions since the currents that can be generated are much lower and thus optimum electrolyte concentrations are far from the diffusion limit.^12^ In addition, Michaels *et al.* demonstrate that a focus on reducing recombination is key to maximise device performance. Furthermore, the light spectrum of indoor light is different from sunlight which give dye-cells an additional edge: the absorption spectrum can be tweaked by co-sensitisation strategies^13^ allowing a much better match to the incoming light spectrum. Here, the team has used sustainable materials in the form of organic dyes and indoor-optimized copper complexes, enabling an extremely high power conversion efficiency of 38%. This phenomenal efficiency is made possible through a novel approach to charge transport materials development, specifically tailored for ambient conditions.

In conclusion, Dr Freitag's team has capitalised on the huge advantages brought using dye-cells for indoor photovoltaics, from tweaking the absorption spectrum through co-sensitisation strategies to developing novel copper-based redox shuttles tailored for indoor conditions, delivering a record-breaking 38% power conversion efficiency. The team has furthermore demonstrated that these can be used to effectively power autonomous, power-adapting IoT devices, capable of sensing and communicating, which is a critical milestone and proof that light harvesting has the potential to effectively power the IoT. Ultimately, the computational capability of the devices is limited by the available energy, so materials developments such as those accomplished by Freitag's team are necessary to push IoT systems beyond the state of the art.

## Data availability

There is no additional data associated with this manuscript.

## Author contributions

Pablo Docampo performed all tasks related to the writing and preparation of the article.

## Conflicts of interest

There are no conflicts to declare.

## Supplementary Material
